# Development of a new monoclonal antibody-based point-of-care testing assay for the quantification of procalcitonin in whole blood for a rapid sepsis diagnostic

**DOI:** 10.1186/cc12913

**Published:** 2013-11-05

**Authors:** Martin Rieger, Daniela Rascher, Anton Hartmann

**Affiliations:** 1 Helmholtz Zentrum München, German Research Center for Environmental Health (GmbH), Department of Environmental Science, Research Unit Microbe-Plant Interactions, Neuherberg, Germany

## Background

After recent studies of the BMBF (SepNet), sepsis causes about 150 deaths per day in Germany, making it the third leading cause of death in Germany. In acute sepsis, rapid diagnosis and rapid medication is crucial. Both as a reliable parameter for diagnosis of sepsis and for guiding the antibiotic therapy, procalcitonin (PCT) is a very sensitive available biomarker [1] and is recommended in the current guidelines [2] to be quantified under sepsis suspicion. Although there are a couple of commercially available fast analytical devices for the quantification of PCT, none of these devices completely fulfill all requirements for a point-of-care testing (POCT) device which are: bedside testing; no sample preparation (whole blood testing); simple handling with ready-to-use and single-use cartridges; and short turnaround time between analysis and medical treatment in the clinical necessary concentration range. Whereas most devices fulfill the latter requirements they are still too big for bedside testing or cannot handle whole blood.

## Materials and methods

Based on newly developed monoclonal antibodies (mAbs) [3], a fast and sensitive immunoassay for the quantification of PCT in whole blood was developed and transferred to a commercially developed (not available on market) POCT device (respons^®^IQ) from pes diagnosesysteme GmbH.

## Results

With the new developed mAbs the achieved limit of detection for PCT in plasma and whole blood is 0.04 ng/ml and 0.05 ng/ml respectively, which is within the clinical necessary range (<0.05 ng/ml). The now established assay shows high reproducibility within 9 minutes, independent of different plasma samples due to the selection of suitable additive compounds. In a first set of leftover patient samples, the PCT-POCT assay showed good correlation (*R*^2 ^= 0.988, *n *= 14, *m *= 2) with the state-of-the-art technology Kryptor (BRAHMs) (D Rascher, M Rieger, HMGU, AMP, unpublished data). Moreover, in cooperation with Dr A Geerlof (HMGU), human recombinant PCT (hrPCT) was produced in two biological and clinical relevant forms (amino acids 1 to 116 and 3 to 116) in high amounts and high purity (A Geerlof, D Rascher, M Rieger, unpublished data). This hrPCT will replace expensive (5 k$/mg) and batch-to-batch varying commercial available hrPCTs as standard reference material.

## Conclusions

The assay shown here for the quantification of PCT fulfils all requirements for POCT. Within 9 minutes, PCT can be quantified near the patient's bed in whole blood without sample preparation.
